# A machine-learning-assisted logistic regression model for predicting post-operative delirium in older adults undergoing total knee arthroplasty

**DOI:** 10.3389/fneur.2026.1828928

**Published:** 2026-07-31

**Authors:** Huajuan Wang, Jie Yu, Lihua Zheng, Ling Xu, Qunyan Zheng, Xing Gao, Wangsheng Wu

**Affiliations:** 1 Department of Anesthesiology, The Quzhou Affiliated Hospital of Wenzhou Medical University (Quzhou People's Hospital), Quzhou, China; 2 Office of Science and Technology Administration, The Quzhou Affiliated Hospital of Wenzhou Medical University (Quzhou People's Hospital), Quzhou, China; 3 Department of Orthopedics, The Quzhou Affiliated Hospital of Wenzhou Medical University (Quzhou People's Hospital), Quzhou, China

**Keywords:** LASSO, machine learning, older adults, post-operative delirium, Random Forest, total knee arthroplasty

## Abstract

**Background:**

Post-operative delirium (POD) is a common complication in older adults undergoing total knee arthroplasty (TKA). Procedure-specific predictive models remain limited. This study aimed to develop a preliminary, internally validated, machine-learning-assisted logistic regression model using routinely available perioperative variables.

**Methods:**

We retrospectively included 451 patients aged ≥60 years undergoing TKA. The cohort was randomly split into a training set (70%) and a validation set (30%). A two-stage selection was applied: Random Forest ranked candidate predictors, and LASSO regression reduced dimensionality. Selected variables were entered into a multivariable logistic regression model to estimate odds ratios (OR) and 95% confidence intervals (CI). Model performance was assessed in the validation set using the area under the receiver operating characteristic curve (AUC).

**Results:**

POD occurred in 137 patients (30.4%). Six variables were selected using Random Forest and LASSO. Logistic regression showed intraoperative hypoxia as the strongest predictor (OR 6.69, 95% CI 2.90–16.6), followed by history of surgery (OR 2.55, 95% CI 1.22–5.32) and higher ASA class (ASA 2: OR 2.31, 95% CI 1.20–4.54; ASA 3: OR 2.01, 95% CI 0.99–4.15). Creatinine (per umol/L) and airway management type also showed smaller associations, while time from admission to surgery was not statistically significant. The model achieved an AUC of 0.704 in the validation set, indicating moderate discrimination.

**Conclusion:**

In older adults undergoing TKA, intraoperative hypoxia, prior surgery, and higher ASA class were associated with POD risk. This preliminary machine-learning-assisted logistic regression model showed moderate discrimination in internal validation and should be externally validated before routine clinical implementation.

## Introduction

Total knee arthroplasty (TKA) is one of the most commonly performed procedures in older adults with advanced knee osteoarthritis, and its use continues to increase with population aging. In this setting, postoperative delirium remains a clinically important complication because it is associated with prolonged hospitalization (1), impaired functional recovery (2), and worse overall postoperative outcomes after joint arthroplasty (3). Systematic reviews and meta-analyses have further shown that delirium is a recurrent and meaningful adverse event after hip and knee arthroplasty in older patients (4–6). Early identification of patients at high risk is therefore of clear clinical importance.

Previous studies have identified a broad range of factors associated with postoperative delirium after total knee arthroplasty and total joint arthroplasty. In TKA-focused cohorts, advanced age, lower educational level, higher ASA class, prior stroke, chronic pulmonary disease, postoperative hypoxemia, longer operative duration, and greater postoperative pain have all been associated with delirium risk (7, 8). Other TKA studies have highlighted sedative-hypnotic exposure and lower preoperative hemoglobin as important predictors in elderly patients (9). In broader arthroplasty populations, cognitive impairment, psychiatric and neurologic comorbidity, nutritional vulnerability, and frailty have also been linked to postoperative delirium (10–13). Overall, the current literature suggests that delirium after arthroplasty reflects the combined effects of baseline vulnerability and perioperative stress.

Despite these advances, many published studies have emphasized individual risk factors or conventional regression-based tools. Earlier screening instruments such as the DEAR tool and short IQCODE established the feasibility of preoperative delirium risk stratification (14, 15), while more recent models such as PROPDESC and PIPRA have improved clinical practicality in broader surgical populations (16, 17). However, relatively few have developed procedure-specific prediction models for geriatric TKA populations (7, 18). Machine learning may help address some of these limitations by integrating multidimensional data and capturing complex non-linear relationships among predictors. In broad perioperative and non-cardiac surgery populations, machine learning models based on routinely collected clinical or electronic health record data have shown promising performance for postoperative delirium prediction (19–21). However, evidence for machine learning-based prediction remains limited in older adults undergoing total knee arthroplasty.

Therefore, this study aimed to develop and internally validate a machine-learning-assisted logistic regression model for predicting postoperative delirium in older adults undergoing total knee arthroplasty. The model used routinely collected perioperative clinical variables and was intended as a candidate risk-stratification tool requiring further external validation.

## Methods

### Study design and participants

This was a retrospective cohort study conducted at The Quzhou Affiliated Hospital of Wenzhou Medical University (Quzhou People's Hospital) between January 2024 and December 2025. Patients who underwent TKA during this period were screened for eligibility. The inclusion criteria were as follows: patients aged 60 years or older who underwent elective primary total knee arthroplasty and had complete perioperative clinical data available in the electronic medical record system. Patients were excluded if they had pre-existing cognitive impairment or psychiatric disorders, a history of delirium before surgery, underwent revision knee arthroplasty, and incomplete clinical or laboratory data (*n* = 13). Therefore, a complete-case analysis was performed, and 451 patients were ultimately included in the final analysis. The study was approved by the Institutional Review Board of Quzhou People's Hospital (Ethical Approval Number: 2025-034), and the requirement for informed consent was waived due to the retrospective nature of the data collection.

### Variables

Preoperative variables included demographic characteristics (age, sex, education level, and marital status), lifestyle factors (smoking and alcohol consumption), and medical history (diabetes, hypertension, hyperlipidemia, coronary heart disease, stroke, and surgery history). Preoperative clinical indicators included time from admission to surgery, preoperative hospital stay, preoperative preparation time, and laboratory parameters such as fasting glucose, LDL, HDL, triglycerides, total cholesterol, albumin, hemoglobin, white blood cell count, red blood cell count, neutrophils, lymphocytes, platelets, C-reactive protein, creatinine (umol/L), and blood urea nitrogen.

Intraoperative variables included operation duration, anesthesia duration, type of general anesthesia (endotracheal intubation or laryngeal mask airway), ASA physical status classification, blood loss, fluid infusion, mean arterial pressure, and intraoperative events. Intraoperative hypoxia was defined as peripheral oxygen saturation < 90% for at least 1 min, intraoperative hypotension as mean arterial pressure < 65 mmHg or a >20% decrease from baseline requiring intervention, and hypothermia as core body temperature < 36.0 C, according to anesthesia record documentation.

### Outcome

The primary outcome was POD within the first seven days after total knee arthroplasty. POD was identified using the Confusion Assessment Method (CAM) for patients on the surgical wards and the CAM-ICU for patients admitted to the intensive care unit (22), based on electronic medical record documentation. Assessments were typically recorded twice daily, at approximately 11:00 and 17:00, by ward or ICU clinical staff who had received institutional training in delirium screening, with physician confirmation when delirium was suspected. For patients discharged before the end of the seven-day observation period, trained study staff conducted telephone follow-up with patients or family caregivers to screen for acute onset or fluctuation in mental status, inattention, disorganized thinking, and altered level of consciousness. A diagnosis of POD was made if CAM criteria were met, requiring acute onset and fluctuating course, inattention, and either disorganized thinking or altered level of consciousness. Because post-discharge assessment was based on pragmatic telephone follow-up rather than direct in-person evaluation, possible outcome misclassification was considered in the limitations.

### Statistical analysis

Categorical variables were presented as frequencies and percentages. Continuous variables were summarized as mean +/- standard deviation when approximately normally distributed, and as median (interquartile range) when distribution was non-normal. Normality was assessed using the Shapiro-Wilk test. Baseline comparisons between groups were performed using the chi-square test or Fisher's exact test for categorical variables, and the independent *t*-test or Mann-Whitney *U*-test for continuous variables, as appropriate based on data distribution. Baseline comparisons between groups were performed for descriptive purposes only and were not used to determine candidate predictors for model development. Categorical variables were presented in grouped form for descriptive purposes, whereas model variables were entered according to their original coding structure in the regression analysis. Variable selection was conducted through a two-stage machine-learning-assisted pipeline designed to distill the most informative predictors from an initial pool of 44 candidate variables (23, 24). First, a Random Forest (RF) model was used to rank all candidate predictors (25). Predictors with positive permutation importance scores were considered potentially informative and retained for subsequent analysis. Second, these variables were entered into a Least Absolute Shrinkage and Selection Operator (LASSO) regression model for dimensionality reduction. The optimal penalty parameter (lambda) was primarily selected using 10-fold cross-validation based on the minimum cross-validated error (lambdamin) to maximize predictive performance. In addition, a sensitivity analysis using the more conservative lambda1se criterion was performed to assess model robustness and parsimony (26). Coefficients that were shrunk to exact zero were interpreted as the absence of any independent predictive signal, and the remaining non-zero terms were retained.

The variables selected by LASSO were then incorporated into a multivariable logistic regression model. Thus, the final predictive engine was an interpretable logistic regression model informed by machine-learning-based variable selection, rather than a complex black-box machine-learning algorithm. We calculated the odds ratios (OR) and 95% confidence intervals (CI) for each predictor. The total cohort was randomly divided into a training set (70%) and a validation set (30%) to develop and evaluate the predictive model. Sample size adequacy for model development was assessed using the events-per-variable (EPV) ratio in the training set. Because approximately 70% of the 137 POD events were allocated to the training set, about 96 events were available for the six final predictors, corresponding to approximately 16 events per variable, which was considered adequate for preliminary model development. The predictive performance of the final logistic regression model was assessed in the independent validation set using the area under the receiver operating characteristic (ROC) curve (AUC) (27). Bootstrap internal validation with 1,000 resamples was also used to estimate optimism and obtain an optimism-corrected AUC. The apparent AUC refers to model performance in the training data before correction, the optimism-corrected AUC refers to bootstrap-corrected internal performance, and the validation AUC refers to performance in the independent 30% validation set. Decision curve analysis (DCA) was performed to evaluate potential clinical utility by quantifying the net benefit across a range of threshold probabilities. Net benefit was compared with default strategies of “treat-all” and “treat-none.” Multicollinearity among predictors retained in the final multivariable logistic regression model was assessed using variance inflation factors (VIFs). For categorical predictors with multiple degrees of freedom, generalized VIFs (GVIFs) were calculated and interpreted as GVIF^∧^(1/(2xDf)).

All statistical analyses were performed using R software (version 4.1.2). Key packages included randomForest, glmnet, rms, pROC, and rmda. *P* < 0.05 was set as the threshold for statistical significance.

## Results

### Baseline characteristics

A total of 451 patients were included, with 137 (30.4%) experiencing postoperative delirium. Patients in the delirium group were significantly older (*P* = 0.035) and had higher mean creatinine levels (*P* < 0.001) compared to the non-delirium group. Significant differences were also found in surgery history (*P* < 0.001) and intraoperative hypoxia incidence (*P* < 0.001). Other characteristics, such as sex, BMI, and most comorbidities, showed no significant differences between groups (Table 1).

**Table 1:** Baseline characteristics of elderly patients undergoing total knee arthroplasty.

Characteristics	Delirium		*P* value
	No	Yes	
*n*	314	137	
Age (mean (SD))	70.19 (6.00)	71.47 (5.70)	0.035
Sex (%)			
Female	202 (64.3)	91 (66.4)	0.748
Male	112 (35.7)	46 (33.6)	
Education (%)			
Primary school or below	111 (35.4)	57 (41.6)	0.388
Junior high school	102 (32.5)	36 (26.3)	
High school/Technical school	32 (10.2)	14 (10.2)	
College	49 (15.6)	17 (12.4)	
Bachelor or above	20 (6.4)	13 (9.5)	
Marital status (%)			
Widowed	1 (0.3)	0 (0.0)	0.558
Married	311 (99.0)	135 (98.5)	
Single	2 (0.6)	2 (1.5)	
Smoking (%)	60 (19.1)	26 (19.0)	1
Alcohol (%)	70 (22.3)	27 (19.7)	0.624
Diabetes (%)	30 (9.6)	11 (8.0)	0.734
Hypertension (%)	70 (22.3)	32 (23.4)	0.9
Hyperlipidemia (%)	74 (23.6)	27 (19.7)	0.435
Coronary heart disease (%)	35 (11.1)	21 (15.3)	0.279
Stroke (%)	37 (11.8)	18 (13.1)	0.804
Time from admission to surgery (days), mean (SD)	2.12 (0.46)	2.18 (0.52)	0.188
Preoperative hospital stay [mean (SD)]	2.08 (0.31)	2.12 (0.40)	0.292
Fasting glucose [mean (SD)]	5.03 (1.18)	4.89 (1.21)	0.275
LDL [mean (SD)]	2.55 (0.70)	2.56 (0.67)	0.95
HDL [mean (SD)]	2.00 (0.97)	1.87 (1.01)	0.183
Triglycerides [mean (SD)]	1.80 (0.85)	1.67 (0.80)	0.137
Total cholesterol [mean (SD)]	4.55 (1.73)	4.36 (1.44)	0.255
Albumin [mean (SD)]	36.46 (1.80)	36.36 (1.67)	0.588
Hemoglobin [mean (SD)]	113.63 (11.32)	113.54 (10.94)	0.937
WBC [mean (SD)]	4.82 (0.77)	4.89 (0.74)	0.335
RBC [mean (SD)]	5.02 (0.41)	5.04 (0.42)	0.6
Neutrophils [mean (SD)]	5.38 (1.27)	5.54 (1.33)	0.223
Lymphocytes [mean (SD)]	3.52 (1.04)	3.53 (1.19)	0.933
Platelets [mean (SD)]	184.95 (44.87)	183.80 (42.74)	0.799
CRP [mean (SD)]	11.01 (8.01)	11.17 (7.47)	0.841
Creatinine (umol/L), mean (SD)	71.61 (23.63)	81.32 (21.81)	< 0.001
BUN [mean (SD)]	4.84 (1.79)	4.92 (1.74)	0.644
Preoperative preparation time [mean (SD)]	2.08 (0.32)	2.12 (0.40)	0.301
Operation duration [mean (SD)]	102.45 (14.23)	102.48 (14.64)	0.984
Surgery history (%)	25 (8.0)	35 (25.5)	< 0.001
Blood loss [mean (SD)]	106.27 (11.41)	106.42 (11.68)	0.899
Intraoperative hypothermia (%)	43 (13.7)	19 (13.9)	1
Fluid infusion [mean (SD)]	1,000.00 (0.00)	1,000.00 (0.00)	/
Intraoperative hypoxia (%)	14 (4.5)	32 (23.4)	< 0.001
Mean arterial pressure [mean (SD)]	82.48 (10.31)	83.50 (10.95)	0.344
Anesthesia duration [mean (SD)]	125.73 (5.14)	126.13 (4.89)	0.442
General anesthesia type (%)			0.110
Endotracheal intubation	214 (68.2)	82 (59.9)	
Laryngeal mask airway	100 (31.8)	55 (40.1)	
ASA class (%)			0.201
1	112 (35.7)	38 (27.7)	
2	116 (36.9)	61 (44.5)	
3	86 (27.4)	38 (27.7)	
Intraoperative hypotension (%)	87 (27.7)	38 (27.7)	1.000

### Variable selection

**Figure 1:**
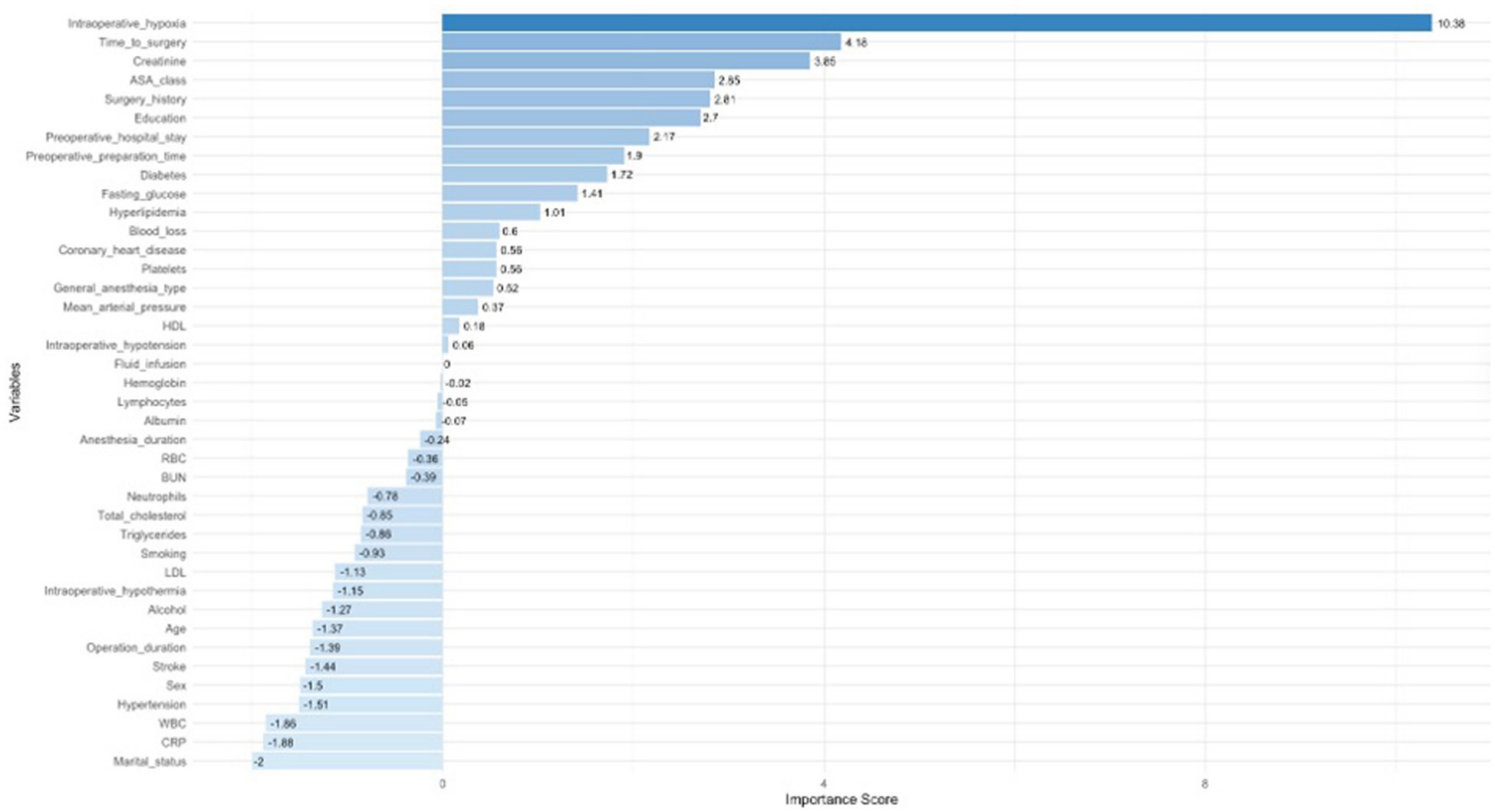
Variable importance of perioperative predictors for post-operative delirium in the training set according to Random Forest analysis.

The RF model applied to the training set initially evaluated all 44 candidate perioperative variables for their contribution to POD prediction. Among these, 18 variables exhibited positive permutation importance scores, indicating a positive impact on the model's predictive performance (Figure 1). The top-ranking variables were intraoperative hypoxia (importance = 10.38), time from admission to surgery (importance = 4.18), and preoperative creatinine (importance = 3.85), followed by ASA class, history of previous surgery, and education level. Other moderately important variables included preoperative hospital stay, preoperative preparation time, diabetes, fasting glucose, hyperlipidemia, blood loss, coronary heart disease, platelets, general anesthesia type, mean arterial pressure, and HDL cholesterol, while intraoperative hypotension and fluid infusion showed minimal contribution.

**Figure 2:**
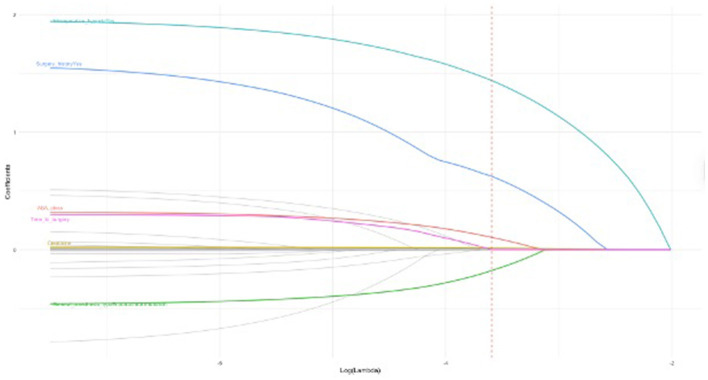
LASSO regression coefficient paths for candidate predictors identified by Random Forest. Each line represents the trajectory of a variable's coefficient across different penalty values (log λ). Red dashed line indicates optimal Lambda (min).

These contributing variables were then subjected to LASSO regression for dimensionality reduction within the training set. Six variables with non-zero coefficients were retained for final modeling: history of previous surgery, time from admission to surgery, preoperative creatinine, ASA class, general anesthesia type, and intraoperative hypoxia (Figure 2). In a sensitivity analysis using the lambda1se criterion, fewer predictors were retained, with modestly reduced discrimination performance. The consistency of the principal predictors across both approaches supported the robustness of the variable-selection process. Using the training set only, approximately 96 POD events were available for the six final predictors, yielding an EPV of approximately 16.0, which was considered adequate for preliminary model development. These variables represented a combination of preoperative patient characteristics, intraoperative events, and anesthesia-related factors, reflecting the multifactorial nature of POD risk.

### Regression model and performance

**Figure 3:**
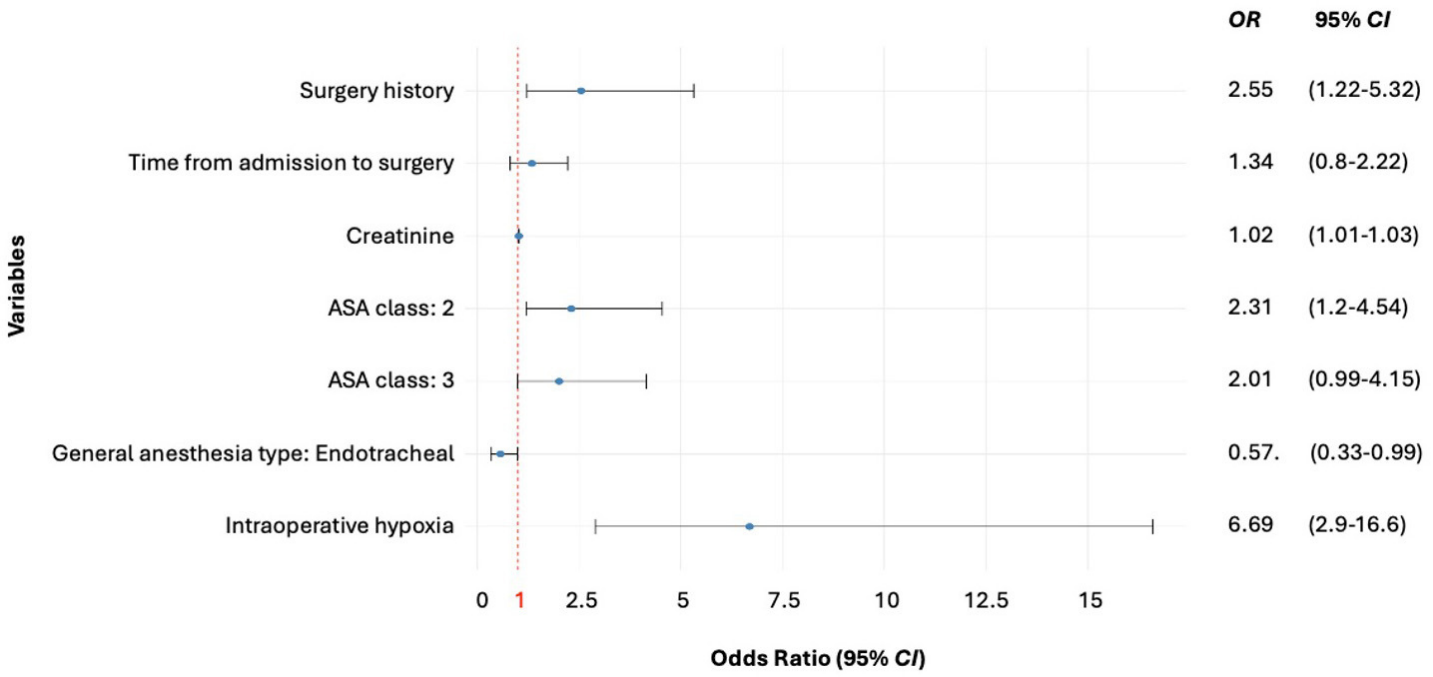
Multivariable logistic regression results presented as a forest plot showing adjusted odds ratios (ORs) and 95% confidence intervals (CIs) for independent predictors of postoperative delirium in elderly patients undergoing total knee arthroplasty.

The six predictors identified by LASSO were incorporated into a multivariable logistic regression model to quantify their associations with POD risk (Figure 3). Intraoperative hypoxia showed the strongest association, with an OR of 6.69 (95% CI: 2.90–16.6). History of previous surgery was associated with higher POD risk (OR 2.55; 95% CI: 1.22–5.32), while ASA class 2 showed an OR of 2.31 (95% CI: 1.20–4.54) and ASA class 3 an OR of 2.01 (95% CI: 0.99–4.15) compared with ASA class 1. Time from admission to surgery had a modest, non-significant association (OR = 1.34; 95% CI: 0.80–2.22). Preoperative creatinine levels showed a small but statistically significant association (OR = 1.02 per umol/L; 95% CI: 1.01–1.03), and endotracheal intubation compared with laryngeal mask airway was associated with lower odds of POD (OR = 0.57; 95% CI: 0.33–0.99). These findings should be interpreted as associations rather than evidence of causality.

**Figure 4:**
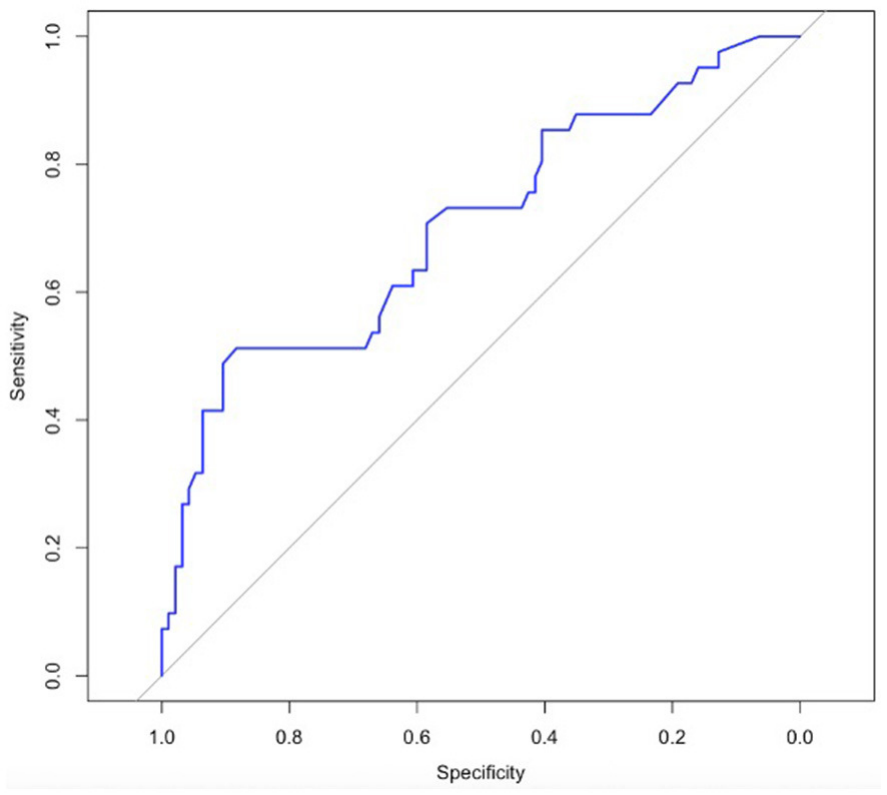
Receiver operating characteristic (ROC) curve of the final logistic regression model in the validation set.

**Figure 5:**
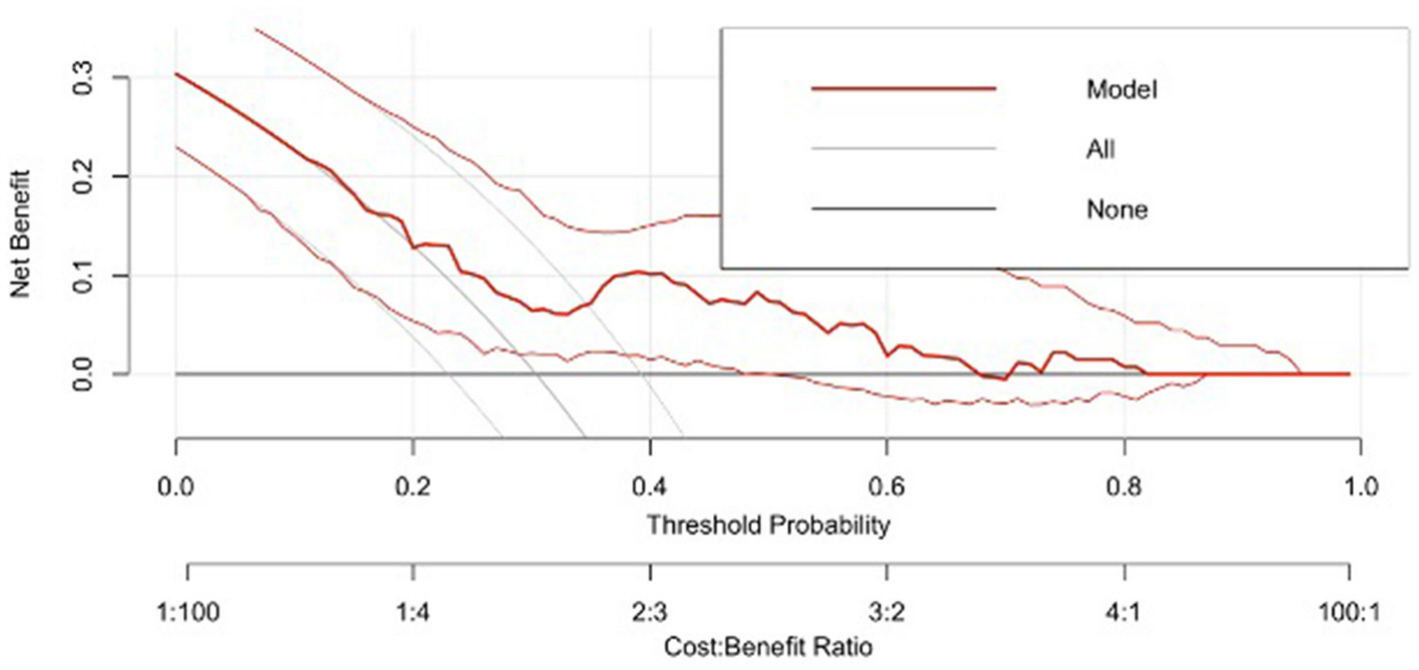
Decision curve analysis evaluating the clinical net benefit of the postoperative delirium prediction model in the validation set.

The predictive performance of the final model was evaluated in the independent validation set. The AUC was 0.7038 (Figure 4), indicating moderate discrimination between patients with and without POD. The calibration intercept was −0.77, and the calibration slope was 1.26, indicating that the model tended to overestimate the risk of POD in the validation group; recalibration would be needed before any future clinical use. The Brier score was 0.182, indicating moderate overall prediction accuracy. Decision curve analysis demonstrated that the predictive model provided a higher net benefit than the “treat-all” and “treat-none” strategies across a range of threshold probabilities, suggesting potential value for perioperative risk stratification (Figure 5). Bootstrap internal validation (1,000 resamples) demonstrated an optimism-corrected AUC of 0.704, compared with an apparent AUC of 0.755, yielding an optimism of approximately 0.051. Assessment of multicollinearity showed very low correlation among final predictors, with VIF/GVIF-adjusted values ranging from 1.006 to 1.032 (Table 2), indicating no evidence of problematic multicollinearity. Overall, the model should be regarded as a preliminary internally validated candidate model that requires external validation before implementation.

**Table 2:** Multicollinearity assessment of predictors in the final logistic regression model.

Predictor	VIF/Adjusted GVIF^a^
Surgery history	1.024
Time from admission to surgery	1.032
Creatinine	1.018
ASA class	1.009
General anesthesia type	1.012
Intraoperative hypoxia	1.006

^a^For categorical predictors with multiple degrees of freedom (ASA class), generalized VIF was transformed as GVIF^∧^(1/(2 × Df)). Lower values indicate less multicollinearity. Values >5 are commonly considered indicative of problematic collinearity.

## Discussion

In this retrospective cohort study of older adults undergoing total knee arthroplasty, we developed and internally validated a machine-learning-assisted logistic regression model for POD. By applying a two-stage variable-selection approach, we identified six perioperative predictors from an initial pool of 44 candidate variables. Multivariable logistic regression showed that intraoperative hypoxia had the strongest association with POD, followed by prior surgery and higher ASA class, while creatinine and airway management type had smaller associations. Time from admission to surgery was not significantly associated with POD. These findings suggest that routinely collected perioperative variables may help identify patients at increased risk, but the model remains preliminary and should be externally validated before being used to guide clinical decision-making.

Intraoperative hypoxia showed the strongest association with postoperative delirium in our cohort. This finding is supported by perioperative literature suggesting that oxygenation-related physiological disturbances are linked to postoperative neurocognitive vulnerability. A recent study in older surgical patients demonstrated a dose-dependent association between intraprocedural hypoxaemia or hypocapnia and postoperative delirium (28). Likewise, intraoperative hypocapnia has been identified as an independent predictor of delirium severity, reinforcing the possibility that disturbed respiratory homeostasis may contribute to acute postoperative brain dysfunction (29). In arthroplasty settings, decreases in regional cerebral oxygen saturation during hip arthroplasty have also been associated with delirium in older adults (4), and a meta-analysis further supported the relationship between perioperative cerebral oxygen desaturation and postoperative delirium in elderly patients (20). Our results extend these observations to older adults undergoing total knee arthroplasty and suggest that intraoperative hypoxia may be a useful marker of perioperative vulnerability. However, because this was an observational study, hypoxia should be interpreted as an associated factor rather than a proven causal driver. It may also act as a composite marker of pulmonary vulnerability, airway difficulty, anesthetic depth, or overall perioperative instability.

The association between higher ASA class and postoperative delirium is consistent with prior evidence, whereas the role of previous surgery history appears more exploratory. Across elective surgery populations, higher ASA class has repeatedly been retained in prognostic models and meta-analyses as a marker of delirium risk (30–32). This is biologically plausible because ASA status captures global preoperative frailty, multimorbidity, and impaired physiological reserve, all of which may increase vulnerability to perioperative stress. By contrast, previous surgery history has not been routinely emphasized in major delirium reviews or prediction tools, which have more often focused on age, cognition, frailty, comorbidity burden, medication use, and prior delirium rather than prior operative exposure itself (31, 33). A history of previous surgery may reflect cumulative surgical burden, repeated anesthetic exposure, more advanced underlying disease, or greater local complexity at the operative site. Because direct supporting evidence remains limited, this result should be interpreted as a potentially important signal that merits confirmation in external datasets.

Elevated creatinine and airway management type showed weaker but still potentially informative associations with postoperative delirium. The creatinine finding is in line with broader delirium literature showing that renal dysfunction contributes to postoperative vulnerability. In prospective presurgical risk modeling, renal failure has been retained as an informative predictor alongside ASA class, multimorbidity, cognition, and surgical duration (30). This supports the interpretation that impaired renal function may contribute to delirium through reduced metabolic reserve, systemic inflammation, toxin accumulation, and greater susceptibility to perioperative physiological derangement. The airway finding is more exploratory. Most prior anesthesia-related delirium studies have compared regional with general anesthesia, or examined broader issues such as anesthetic depth and perioperative cognitive outcomes, rather than directly comparing endotracheal intubation with laryngeal mask airway (34, 35). Therefore, this association should not be regarded as definitive or causal. It may reflect differences in patient selection, anesthetic management, airway strategy, or unmeasured perioperative risk rather than the independent effect of the airway device itself.

The model's clinical applicability is promising but not yet established. The use of routinely available variables may facilitate future translation, and the logistic regression format preserves interpretability. However, the validation AUC of approximately 0.70 indicates moderate discrimination, and the present analysis was based on a single-center retrospective cohort with internal validation only. Accordingly, the model should be viewed as an early candidate risk-stratification tool. Prospective external validation, recalibration in independent populations, and assessment of whether model-guided interventions improve patient outcomes are needed before routine clinical implementation.

There are several limitations. First, patients with missing key variables were excluded and complete-case analysis was used, which may have introduced selection bias if missingness was not completely random. However, the proportion of excluded patients due to missing data was low (< 5%). Second, the present study was conducted in a single-center retrospective cohort without external validation, which may limit generalizability. Third, although POD was assessed using CAM or CAM-ICU documentation, delirium is a fluctuating syndrome and retrospective ascertainment may miss short or undocumented episodes. Post-discharge delirium was captured by telephone follow-up when needed, which may introduce recall and information bias because direct in-person assessment was not available. Fourth, several important baseline vulnerability factors, including detailed preoperative cognitive testing, frailty assessment, medication exposure, sleep quality, pain trajectory, and prior delirium history, were unavailable or incompletely captured in the retrospective dataset. Omission of these variables may partly explain the model's moderate predictive performance. Finally, the model has not yet been externally validated, prospectively tested, or incorporated into a clinical decision tool; therefore, it should not be considered ready for routine clinical implementation.

## Conclusion

In this retrospective cohort study of older adults undergoing total knee arthroplasty, we developed a preliminary machine-learning-assisted logistic regression model for postoperative delirium. Intraoperative hypoxia, history of previous surgery, higher ASA class, preoperative creatinine, and airway management type were associated with POD risk. The model demonstrated moderate discrimination in internal validation, suggesting that routinely available perioperative variables may help identify higher-risk patients. External validation and prospective evaluation are required before the model can be recommended for routine clinical use.

## Data Availability

The raw data supporting the conclusions of this article will be made available by the authors, without undue reservation.
